# Study of Defects and Nano-patterned Substrate Regulation Mechanism in AlN Epilayers

**DOI:** 10.3390/nano12223937

**Published:** 2022-11-08

**Authors:** Wenwang Wei, Yi Peng, Yanlian Yang, Kai Xiao, Mudassar Maraj, Jia Yang, Yukun Wang, Wenhong Sun

**Affiliations:** 1 Research Center for Optoelectronic Materials and Devices, School of Physical Science & Technology, Guangxi University, Nanning 530004, China; 2 College of Materials and Chemical Engineering, Hezhou University, Hezhou 542899, China; 3 MOE Key Laboratory of New Processing Technology for Nonferrous Metals and the Guangxi Key Laboratory of Processing for Non-ferrous Metallic and Featured Materials, Guangxi University, Nanning 530004, China

**Keywords:** nano-patterned, AlN, dislocation densities, etch pit density

## Abstract

The high crystal quality and low dislocation densities of aluminum nitride (AlN) grown on flat and nano-patterned sapphire substrate that are synthesized by the metal-organic chemical vapor deposition (MOCVD) method are essential for the realization of high-efficiency deep ultraviolet light-emitting diodes. The micro-strains of 0.18 × 10^−3^ cm^−2^ for flat substrate AlN and 0.11 × 10^−3^ cm^−2^ for nano-patterned substrate AlN are obtained by X-ray diffractometer (XRD). The screw and edge dislocation densities of samples are determined by XRD and transmission electron microscope (TEM), and the results indicate that the nano-patterned substrates are effective in reducing the threading dislocation density. The mechanism of the variation of the threading dislocation in AlN films grown on flat and nano-patterned substrates is investigated comparatively. The etch pit density (EPD) determined by preferential chemical etching is about 1.04 × 10^8^ cm^−2^ for AlN grown on a nano-patterned substrate, which is slightly smaller than the results obtained by XRD and TEM investigation. Three types of etch pits with different sizes are all revealed on the AlN surface using the hot KOH etching method.

## 1. Introduction

Deep ultraviolet light emitting diodes (DUV-LEDs) based on III-nitride semiconductors are expected to be used in sterilization, disinfection, medical treatment and curing [1]. To achieve high efficiency and long-term reliability of DUV-LEDs, it is crucial to improve the crystalline quality of AlGaN films as light-emitting and conducting layers [2]. In addition, the crystallization quality of the AlN buffer layer on the substrate severely affects the crystallization quality of AlGaN [3]. Therefore, in order to improve the performance of DUV-LEDs, AlN templates with high crystalline quality must be realized on heterogeneous substrates. Although many studies have been carried out to improve the quality of AlN films on sapphire, such as the modulation of growth conditions [4], high temperature thermal annealing [5] or epitaxial lateral overgrowth (ELOG) on nano-patterned sapphire (NPSS) [6], the threading dislocation densities (TDDs) of AlN films are still in the order of 10^9^ cm^–2^. Generally, in order to eliminate threading dislocations (TDs), it is necessary to grow a relatively thick AlN epitaxial layer to realize the paired annihilation of TDs [7]. During the heterogeneous epitaxial growth process, large dislocation density and residual stresses are generated between the AlN film and the sapphire substrate due to the difference in lattice constants and thermal expansion coefficients. Many previous studies have shown that the nano-patterned substrate growth methods can effectively reduce the TDD and improve the crystal quality of AlN epitaxial films [8,9,10]. Zhang et al. [8] used a lower rate of lateral growth of AlN on nano-patterned sapphire by the nano-imprinting technique to reduce the misorientation in adjacent regions during merging and to decrease the TDDs. The embedded periodic air-voids formed by the nano-patterned substrate help to improve the crystal quality of the epitaxial layer and break the in-plane propagation of DUV photons, thus increasing the light extraction efficiency [11]. The AlN/NPSS epilayers exhibit compressive stresses rather than tensile stresses due to stress relaxation by key-shaped holes in the substrate, and very low TDD and etch pit density (EPD) were obtained by modulating the V/III ratio alternating growth [9]. In addition, many dislocations in AlN films can be eliminated by introducing an effective in-situ ELOG to obtain low defect density, while NPSS can suppress tensile stresses induced by grain boundary [12]. Long et al. [13] grew high-quality, crack-free and strain-free AlN epilayers with thicknesses over 10 μm on pyramidal sapphire substrates, the dislocation double coalescence process was observed, and additional dislocation annihilation and strain relaxation channels were introduced into AlN epilayers. The TDD of the AlN/sapphire template has reached or is even lower than 10^8^ cm^−2^ in many experiments, but the TDD available in commercial production is generally in the order of 10^9^ cm^−2^ [14]. For a long time, excessive dislocation density has been a concern, mainly because high TDD leads to the reduction of the internal quantum efficiency, which seriously restricts the performance and application of AlN DUV-LEDs. However, there is no uniform standard for the evaluation of TDD. In addition to the measurement method, different devices may also yield different results for dislocation density evaluation. The study of the microstructure and origin of etch pits in GaN epilayers by Lu et al. [15], laid a foundation for the research of nitride materials in etch pits and provided vitally important research ideas. Although many methods for etching AlN have been reported, and it has also been found that alkaline solutions are more reliable for etch pit characterization of AlN, the EPD obtained by researchers are relatively small (10^3^–10^7^ cm^−2^), which cannot accurately reflect the true dislocation density of AlN materials [9,16,17].

In this work, high-quality AlN epilayers were grown on different sapphire substrates by metal-organic chemical vapor deposition (MOCVD). On the one hand, we compared the dislocation density of different substrate samples by various means (such as XRD, TEM and SEM), analyzed the reasons for the differences, and elucidated the mechanism of defect propagation annihilation. On the other hand, the etch pits of AlN epilayers were systematically studied through hot KOH solution. This etching condition could more accurately reflect the dislocation density on the surface of AlN and reveal the process of etching behavior, which enable us to grow high-performance DUV electronic devices and then meet the urgent needs of the industrial production for DUV devices.

## 2. Materials and Methods

AlN samples were grown by MOCVD on a 2-inch [0001]-direction conventional (flat) sapphire substrate and nano-patterned sapphire substrate, namely Sc and Sp, respectively. The growth mode of Sc and Sp are continue mode and continue + pulse mode, respectively. The nano-patterned substrate has 400 nm deep truncated cone patterns with a 550 nm diameter at the top circumference and 650 nm at the bottom. The pitch length is ~1000 nm. The sapphire substrates were coated with ~15 nm AlN layer by physical vapor deposition (PVD). For the epitaxial growth in MOCVD, the precursors of Al and N were trimethyl-aluminum (TMAl) and ammonia (NH_3_), respectively. Hydrogen (H_2_) was the carrier gas. Firstly, the sapphire substrates with a PVD AlN nucleation layer were heated to 1150 °C in H_2_ ambient, followed by the initial AlN roughing layer growth with ten atomic layers. The temperature was then ramped up to 1250 °C and maintained at two-dimensional growth until the end of the film growth. The further details of growth information, thickness, and root mean square (RMS) surface roughness of Sc and Sp have been given in Table 1 [18].

X-ray measurements were performed using a high-resolution X-ray diffractometer (HRXRD, X′Pert^3^ MRD) equipped with a Ge (220) four-crystal monochromator in a triple-axis model (Cu K_α1_ = 1.5406 Å radiation) to investigate the crystal quality of the AlN epitaxial layers, while the TDDs of screw type and edge type were estimated. Transmission electron microscopy (TEM, FEI Talos F200X) investigations were carried out with a 200 keV to explore the development process and evolution mechanism of threading dislocations (TDs). Samples were prepared by mechanical thinning, followed by argon ion-beam thinning (Gatan 695) for cross-sectional high-resolution observations of dislocations with diffraction vectors **g** = [0002] and **g** = $\left[01\bar{1}0\right]$ under the condition of $\left[11\bar{2}0\right]$ zone axis. The small pieces of AlN/sapphire samples placed in a Teflon holder were wet-etched in a KOH solution (5 mol/L) at 80 °C to reveal the etch pits, followed by rinsing in deionized water using an ultrasonic cleaner. The EPD of samples were counted from the surface scanning electron microscope (SEM, Sigma 500) images after etching.

## 3. Results and Discussion

**Figure 1:**
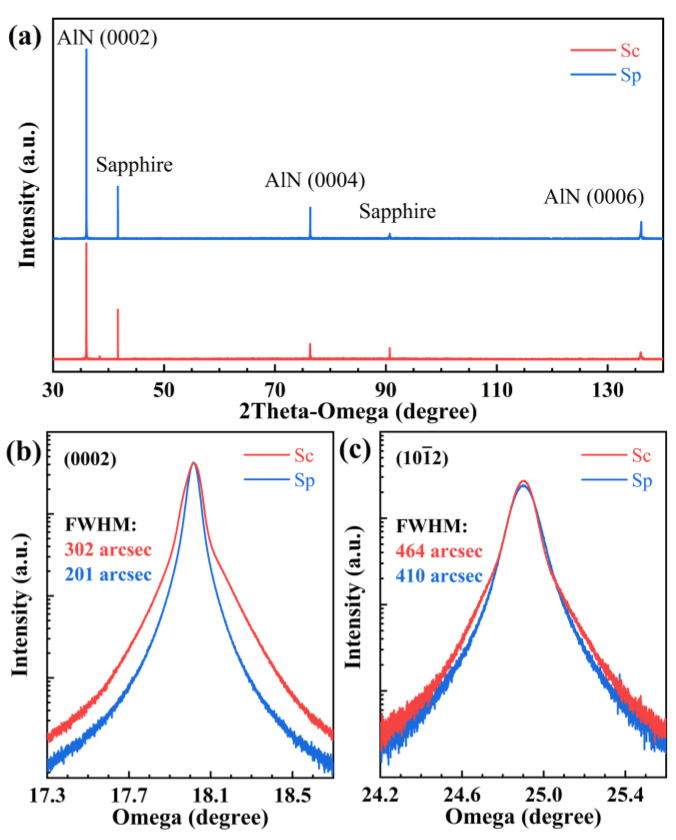
(**a**) The XRD patterns of AlN samples. (**b**) (0002) and (**c**) $\left(10\bar{1}2\right)$ plane X-ray rocking curves of samples.

The crystal quality, micro strain and dislocation density of the AlN epilayers were investigated by HRXRD analysis. The heteroepitaxy c-plane AlN film has a large number of TDs, making the epilayer a mosaic structure composed of multiple sub-grains. The lattice deformation was caused by dislocation, which would give rise to tilt or twist in the hexagonal cells. The dislocation line is in the direction of (0001). The micro strain (ε) can be calculated by using the Stokes–Wilson equation [19].

(1) $$\varepsilon =\frac{\beta }{4tan\theta }$$

where θ is the Bragg diffraction angle and β is the full width at half maxima (FWHM) corresponding the film Bragg peak at 2θ [20]. The HRXRD patterns of Sc and Sp are presented in Figure 1a and the diffraction peaks of AlN (0002), (0004), and (0006) have been observed. The micro strain for Sc and Sp is given as 0.18 × 10^−3^ and 0.11 × 10^−3^, respectively. The results show that the AlN epilayer with patterned substrate has better crystal quality.

The (0002) and $\left(10\bar{1}2\right)$ X-ray rocking curves (XRC) from different substrate samples are shown in Figure 1b,c. The XRC of Sc shows distinct asymmetry and a shoulder at larger omega values; this splitting phenomenon of the diffraction peak could be caused by the polarization of the AlN film. The FWHM of the rocking curves’ symmetric and asymmetric diffraction indirectly reflects the TDs density of different types. The FWHM of symmetric diffraction (0002) is sensitive to the density of pure screw and mixed type TDs, while the FWHM of asymmetric diffraction $(10\bar{1}2)$ can effectively reflect the pure edge and mixed type TDs [21]. According to the FWHM of the rocking curve, TDD can be calculated quantitatively, expressed as:

(2) $$\rho =\frac{\beta }{2\pi b^{2}\ln2}\approx \frac{\beta^{2}}{4.35b^{2}}$$

where *ρ* represents dislocation density, *β* stands for the FWHM of the rocking curve and *b* is the length of Burgers vector of dislocation. The Burgers vector ***b*** for screw dislocation, edge dislocation, and mixed dislocation are $b_{s}=\langle 0001\rangle$, $b_{e}=1/3\langle 11\bar{2}0\rangle \;$, and $b_{m}=1/3\;\langle 11\bar{2}3\rangle$, respectively [22]. The AlN Burgers vector lengths of screw type and edge type TDs are *b_s_* = 0.4982 nm and *b_e_* = 0.3112 nm, respectively. The FWHM of symmetric (0002) rocking curve for Sc and Sp are 302 arcsec and 201 arcsec, respectively. The FWHM of the asymmetric $\left(10\bar{1}2\right)$ rocking curve for Sc and Sp are 464 arcsec and 410 arcsec, respectively. The (0002) and $\left(10\bar{1}2\right)$ FWHM of the Sc films are larger than that of the Sp, revealing that Sp is of higher crystal quality than Sc. The density of screw type and edge type dislocations are estimated and are listed in Table 2. For sample Sc, the screw and edge dislocation density are 1.99 × 10^8^ cm^−2^ and 1.20 × 10^9^ cm^−2^, while those values are as low as 8.80 × 10^7^ cm^−2^ and 9.38 × 10^8^ cm^−2^ for Sp, respectively. The results show that the nano-patterned substrate is beneficial for the reduction of the TDDs and improves thin film crystal quality.

**Figure 2:**
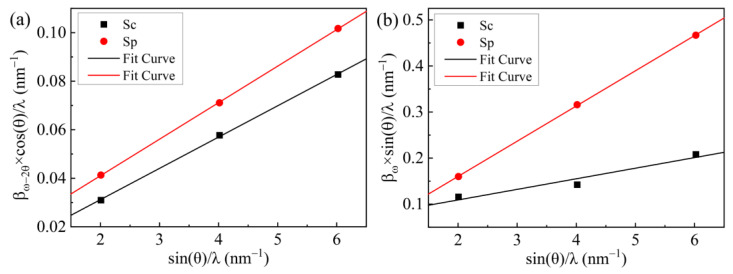
Williamson–Hall plots of AlN films for (**a**) ω–2θ scan and (**b**) ω scan symmetric reflections.

Figure 2 shows the Williamson–Hall plot for the AlN epilayers, where ω scan and ω–2θ scan have been measured for three symmetric reflections: (0002), (0004) and (0006). The corresponding parameters of AlN epilayers are deduced by linear fitting from Figure 2. It can be derived that the lateral coherence length L_//_, vertical coherence length L_⊥_, dislocation tilt angle β_t_ and non-uniform strain $\varepsilon_{\mathrm{in}}^{⊥}$ for Sc are 45.70 nm, 68.40 nm, 0.076°, 0.015, respectively. Those data for Sp are 92.76 nm, 79.20 nm, 0.023°, 0.012, respectively. The results show that the grain size of the Sp sample is larger and the non-uniform strain in the growth direction is smaller.

**Figure 3:**
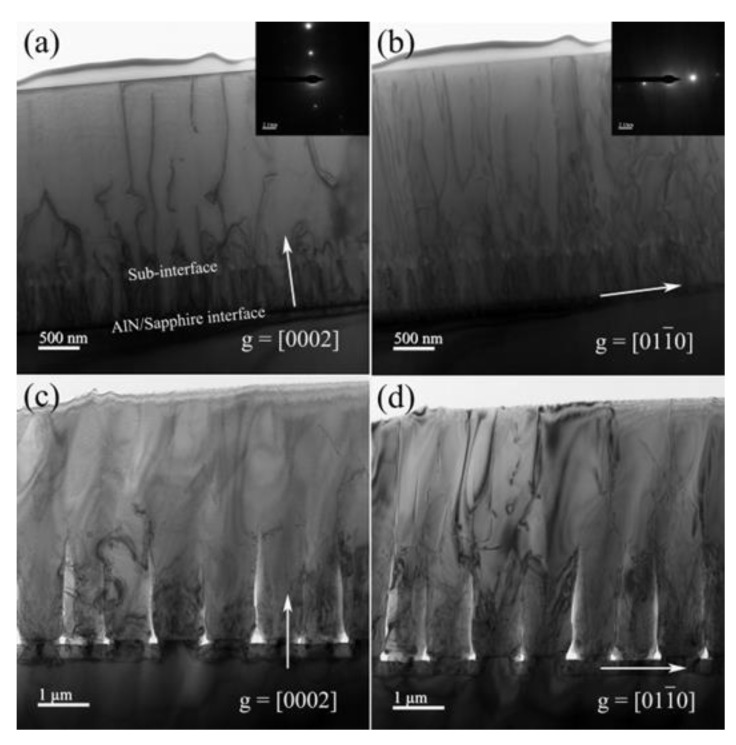
Bright-field cross-sectional TEM images of AlN/Sapphire sample Sc (**a**,**b**) and Sp (**c**,**d**) along with g = [0002] and g = $\left[01\bar{1}0\right]$.

TEM observation is a necessary means of microanalysis, especially for the detection and analysis of internal defects of materials, such as dislocations, phase boundaries, grain boundaries, cracks and cavities, etc. Figure 3 represents cross-sectional bright-field TEM images of Sc and Sp under the two-beam condition with **g** = [0002] and $\left[01\bar{1}0\right]$, respectively. Using the dislocation invisibility criterion of **g · b** = 0, the Burgers vector of the dislocation can be determined, and then the densities of different type dislocations can be calculated [23]. Threading dislocations are characterized as screw type, edge type, and mixed type: screw type dislocations are visible for **g** = [0002], while edge type dislocations are visible for **g** = $\left[11\bar{2}0\right]$, $\left[2\bar{1}\bar{1}0\right]$, $\left[01\bar{1}0\right]$ or $\left[1\bar{1}00\right]$; all types of dislocations are also usually characterized by the **g** = $\left[10\bar{1}1\right]$, $\left[11\bar{2}2\right]$ or $\left[01\bar{1}1\right]$ diffraction vector [24,25,26]. From Figure 3, the density of screw type and edge type dislocations for Sc are evaluated to be 9.15 × 10^8^ cm^−2^ and 3.89 × 10^9^ cm^−2^, while the corresponding values of Sp are estimated to be 4.32 × 10^8^ cm^−2^ and 1.58 × 10^9^ cm^−2^ by observing cross-sectional TEM images, respectively. Thus, the detailed values of different type dislocations density extracted and compared for AlN samples are given in Table 2. It can be seen that the dislocation density on the surface of Sc is higher than that of Sp. Those results are in good accordance with the XRD and EPD findings. In addition, a significant reduction of dislocation lines above the sub-interface of sample Sc can be observed, which is mainly attributed to two cases: (i) the reorientation of the sub-interface, resulting in a large kink in the dislocation lines at the sub-interface and the TD reorients; and (ii) the reorientation of the two TDs to each other, forming a dipole half-loop [26].

**Figure 4:**
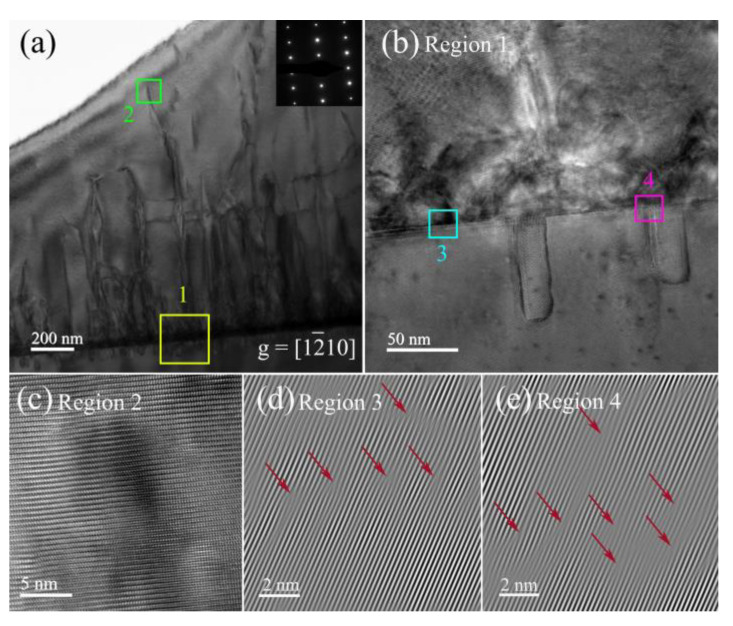
(**a**) Cross-sectional TEM image of AlN sample Sc. (**b**,**c**) Partial enlarged images corresponding to region 1 and region 2 in (**a**). (**d**,**e**) Inverse fast Fourier transform images for region 3 and region 4 in (**a**).

A further detailed analysis of Sc and Sp samples is necessary to understand the development process and evolution mechanism of dislocations in AlN thin films. Figure 4a shows the cross-sectional TEM image of the sample Sc. The distribution of dislocations can be clearly observed under the condition of **g** = $\left[1\bar{2}10\right]$. In the lower half of the AlN epilayer, dislocations are distributed in the form of dislocation bundles, which is conducive to the concentrated annihilation of dislocations. Meanwhile, the bending of dislocations blocks partial dislocations from extending to the surface, which is beneficial to reducing the surface dislocation density. In the upper half of the AlN epilayer, the dislocations can be observed to decrease significantly. Figure 4b is an enlargement of the interface between the substrate and the AlN epilayer. There are a lot of defects in the interface due to large lattice mismatch and thermal mismatch at the initial stage of growth. The high-resolution image after the extension of the TD is shown in Figure 4c. The TDs cause lattice distortion, and dislocation regions have larger d-spacing than nearby dislocation-free regions, indicating compressive stress in the AlN epilayer. In [29], the stress change caused by the change of lattice constant at different depths of the film has been discussed. In order to make the lattice defects more clearly visible, the fast Fourier transform (FFT) of regions 3 and 4 are performed to remove the diffraction spots parallel to the crystal plane in the interface direction, and then Figure 4d,e are obtained by inverse FFT. The lattice mismatch in different regions of the interface can be seen more clearly, and here dislocations are dominated by edge dislocations.

**Figure 5:**
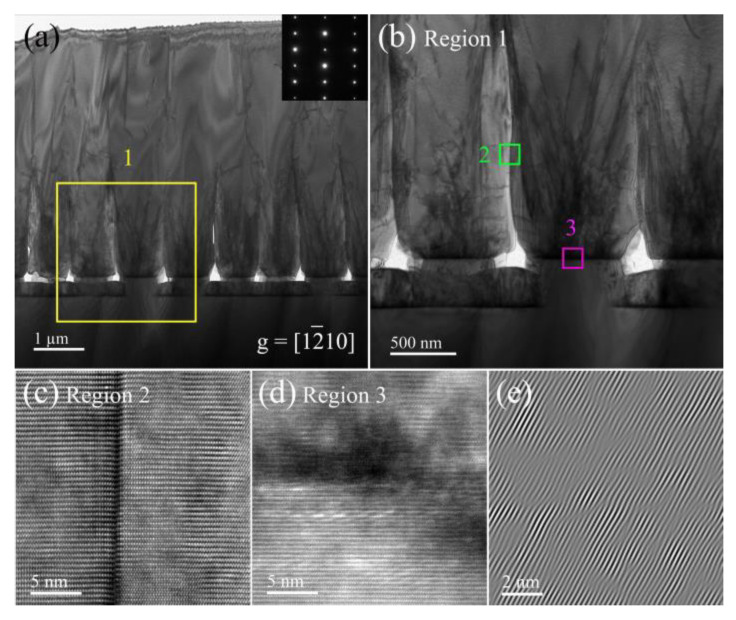
(**a**) Cross-sectional TEM image of AlN sample Sp. (**b**) Partial enlarged image corresponding to region 1 in (**a**). (**c**,**d**) HRTEM images focused on regions 2 and 3. (**e**) Inverse fast Fourier transform image for region 3.

It can be seen from Figure 5a that at the beginning of the growth process, a large number of defects are introduced due to lattice mismatch and thermal mismatch between the substrate and film, forming a dislocation pile-up region and mainly TDs. The TDs are generated by the growth interface and propagate to the surface along the growth direction. As the growth proceeds, the film completely coalesces up to a thickness of ~2 μm from the substrate, and the TDs propagating upward at the closure interface are greatly reduced. The distribution and variation of TDs can be more easily observed in Figure 5b; some dislocations bend or propagate laterally toward free interface before the hole is coalesced, which may be caused by the internal stress. The TDs will preferentially penetrate the areas that are more likely to be exposed. This behavior is beneficial to prevent the TDs propagating to the surface of the film, thus the TDD is effectively reduced. Furthermore, a large fraction of the dislocations is annihilated in the form of fault loops and coalescing dislocations. The decrease in dislocation density is due to the fact that the adjacent dislocations have opposite Burgers vectors forming a loop as the thickness of the epilayer increases [27]. As a result of stress variation near the interface, some TDs could be terminated and not penetrate into the epilayer surface owing to the dislocation line energy minimization [28]. HRTEM measurement is performed to focus on the interface of regions 2 and 3. On the AlN epilayer coalescence interface, we can see the severe lattice mismatch, while dislocations penetrate to the sample surface, as shown in Figure 5c. This is also why the EPD of sample Sp are more likely to be revealed by wet etching. In Figure 5d, the dislocations at the interface are very dense and appear as a black blur, so it is difficult to clearly distinguish the crystal plane. However, by the inverse FFT of Sp interface in Figure 5e, it can be seen that the interface of the nano-patterned substrate has a higher density of misfit dislocations than the flat substrate. The strain could be easily relaxed by the large number of grain boundaries in the hexagonal columnar regions in the process of decreasing temperature, resulting in high-density TDs [29].

**Figure 6:**
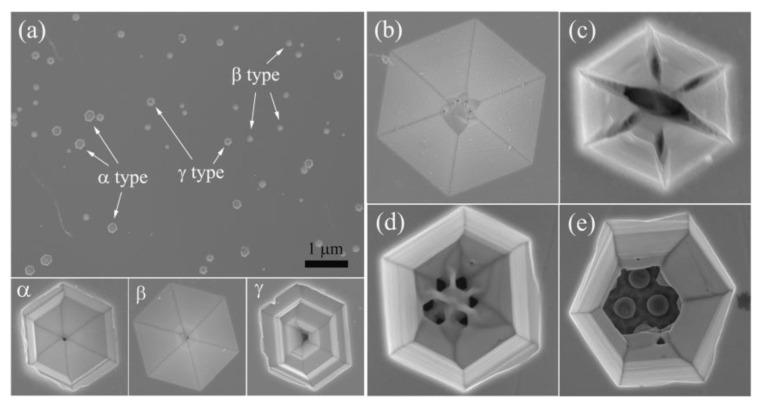
(**a**) SEM images of Sp surface at 80 °C etching by KOH for 12 min. (**b**–**e**) Plan-view SEM images of the β-type dislocation which show the etching evolution of wurtzite AlN surface.

Wet selective etching has a strong corrosive effect on defective surfaces with strain zones and chemical inhomogeneity, such as dislocations and impurity streaks. An aqueous KOH solution of 5 mol/L was used to etch the AlN materials at 80 °C with holding time for 12 min, and the surface morphology after etching was observed by SEM, as shown in Figure 6a. Three different morphologies of etch pits are found, which can be referred to as α-type, β-type, and γ-type etch pits, respectively. The α-type etch pit corresponds to a pure screw dislocation with a spiral stepped structure. As etching proceeds, the chemical stability of the Al surface will prevent further etching in the vertical direction, while the etching rate in the lateral direction remains constant. The β-type etch pit represents pure edge dislocations. Each atom on the edge dislocation line has a dangling bond which is easily etched, so the corrosion proceeds continuously along the vertical dislocation line direction, forming an inverted hexagonal pyramid β-type etch pit. The γ-type etch pits are a combination of the α-type and β-type, combining the characteristics of both, and therefore belong to mixed type dislocation. Under optimal etching conditions, over-time etching resulted in over-etching of the samples and also the merging of etch pits together. From Figure 6a, it can be seen that the size of α-type etch pits is the largest, while the size of β-type etch pits is the smallest, indicating that in the etching process pure screw dislocations are more susceptible to erosion by etchant. Under the same etching conditions, AlN grown on a flat sapphire substrate is less prone to show up etch pits than that on the patterned substrate. This is mainly due to the fact that while patterned substrates can effectively promote the merging and annihilation of dislocations, it also tends to form vertically extending dislocation bundles, which makes them more likely to propagate in the epitaxial layer and thus corrode at the surface outcrop. Details of EPD for both AlN samples are listed in Table 2. The etch pit densities of the Sc and Sp samples are 1.23 × 10^9^ cm^−2^ and 1.04 × 10^8^ cm^−2^, respectively. It can be seen that the growth of the AlN epilayer on the nano-patterned substrate is beneficial to reduce the TDDs and improve the crystal quality of the AlN epilayer.

It is of great theoretical and practical significance to understand the etching mechanism of wurtzite AlN surface texturing or selective etching. Figure 6b–e shows the plan-view SEM images of the β-type dislocation etch pits on the AlN surface. As shown in Figure 6b, the etch pit shows a typical inverted hexagonal pyramid-shaped with an Al-polarity plane and randomly distributed on the surface during the initial stage of etch. However, N-polarity AlN crystals showed hexagonal hillocks after etching in a hot KOH solution [30]. Figure 6c illustrates that the junction of each surface in the hexagonal pit begins to split and is further etched, which accelerated the etch rate in the vertical direction. Figure 6d reveals the formation of new holes within the etch pit and the penetration of the etchant into the AlN film through these holes. With the increase of time, the etch pits have been over-etched to the substrate in the vertical direction, and the nano-patterned columns are clearly visible, as shown in Figure 6e. The appearance of etch pits is related to the surface energy: the dangling bonds density and the planar density determine the surface energy, and the outcrop of the dislocation surface of the film is selectively etched by high-energy etchant, while the rest of the area remains intact [31]. As the etching proceeds, the etch pits grow in size, and the boundaries of adjacent etch pits gradually disappear and merge into one larger etch pit. The surface energy is reduced and the stability is improved by the interface combining between the etch pits [32]. To a certain extent, this behavior slows down the etch process and plays a certain role in the self-protection of the film.

**Figure 7:**
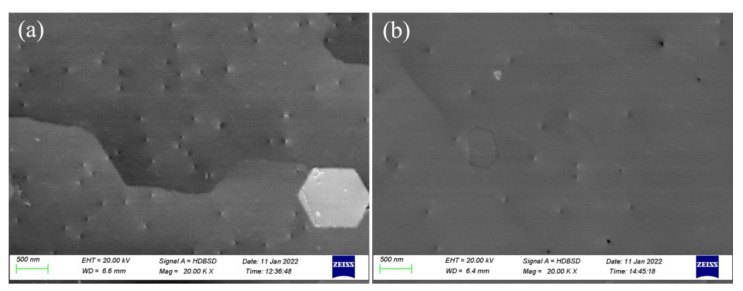
Electron channel contrast imaging (**a**) sample Sc and (**b**) sample Sp.

Figure 7 shows the characterization of large area and high flux crystal defects by using electron channel contrast imaging (ECCI) technology of SEM. The basic principle is that there will be an abrupt change in the yield of backscattered electrons when the incidence angle θ of the electron beam relative to the lattice orientation is around the Bragg diffraction condition θ_B_ (an abnormal increase for θ < θ_B_ and an abnormal decrease for θ > θ_B_). After introducing defects into AlN crystal materials, the atomic arrangement in the crystal will change, and the yield of backscattered electrons near the defects will also change. In Figure 7, it can be clearly observed that there are significantly fewer defects penetrating the surface in sample Sp than that of Sc, which further illustrates that there are fewer defects in the sample grown on the nano-patterned substrate. In addition, the defects are mainly divided into convex and concave. Edge threading dislocations always occupy the majority in the material. Therefore, it can be judged that a large number of concave defects are edge type dislocations and a relatively small number of convex defects are screw-type dislocations.

At present, XRC scanning is the most commonly used method to evaluate the TDD. However, the penetration depth of X-ray in AlN material is large, i.e., the measurement results contain the information of the entire AlN epilayer and not just the top information of the epilayer surface. The TDD of the surface of the AlN epilayer can be obtained from cross-sectional TEM and plan-view TEM, but they have a certain randomness as the thickness of TEM sample is less than 100 nm and the measured area is usually less than 10 μm^2^. The cross-sectional TEM technique for defect density quantification is limited to a TDD of at least 10^8^ cm^−2^. The TDD assessed by EPD is also inaccurate when the AlN surface is over- or under-etched. Furthermore, different equipment may give different results even if the measurement method is the same [33]. Even so, the TDD obtained through specific analysis are also representative, which can provide a valuable reference for understanding the overall development trend of AlN materials.

## 4. Conclusions

The crystal quality, micro-strain, and dislocation density of the AlN epilayers on flat sapphire substrate (Sc) and nano-patterned sapphire substrate (Sp) have been investigated. The micro-strain of 0.18 × 10^−3^ cm^−2^ for Sc and 0.11 × 10^−3^ cm^−2^ for Sp is obtained. For the XRC data, the screw dislocation density difference for Sc and Sp is roughly a factor of two, and it is even less for edge dislocations. It is also approximately a factor of two for the TEM data. The results indicate that the nano-patterned substrates are effective in reducing the TDD. The TDs in AlN on nano-patterned substrates extend more easily to the surface, but there is a smaller dislocation density, and such a result is also confirmed in the EPD experiments. The TDs preferentially penetrate to the surface where they are easily exposed, except for annihilation by fault loops and dislocation coalescing, etc. Finally, etch pits of three different morphologies, α-type, β-type and γ-type, are obtained by selective etching of the surface of AlN with aqueous KOH solution. The EPD of 1.23 × 10^9^ and 1.04 × 10^8^ cm^−2^ are determined for Sc and Sp, respectively. The defective areas on the material surface are susceptible to the etch effect.

## Figures and Tables

**Table 1:** Summary of the key aspects for AlN samples.

Sample No.	Growth Mode	Precursor	AlN Thickness (µm)	RSM (nm)
Sc	Continue	TMAl, NH_3_	3.02	2.32
Sp	Continue + Pulse	TMAl, NH_3_	5.12	2.36

**Table 2:** The dislocation density obtained by XRD and TEM, and the etch pit density of the AlN samples.

Sample No.	Dislocation Density (cm^−2^) by XRD		Dislocation Density (cm^−2^) by TEM		Etch Pit Density (cm^−2^)
	Screw Type	Edge Type	Screw Type	Edge Type	
Sc	1.99 × 10^8^	1.20 × 10^9^	9.15 × 10^8^	3.89 × 10^9^	1.23 × 10^9^
Sp	8.80 × 10^7^	9.38 × 10^8^	4.32 × 10^8^	1.58 × 10^9^	1.04 × 10^8^

## Data Availability

The data presented in this study are available on request from the corresponding author.
